# Mechanism and Active Components of Qingre Lidan Tablets Alleviate Intrahepatic Cholestasis by Activating the Farnesoid *X* Receptor

**DOI:** 10.1155/2022/1589388

**Published:** 2022-11-30

**Authors:** Yafei Xia, Shixin Yan, Yang Chang, Jiaoyang Yin, Hongyu Li, Shu Yan

**Affiliations:** ^1^Tianjin Medical University Nankai Hospital, No. 6 Changjiang Road, Nankai District, Tianjin 300100, China; ^2^Tianjin Nankai Hospital, No. 6 Changjiang Road, Nankai District, Tianjin 300100, China; ^3^Beijing Fukangren Biopharmaceutical Co., Ltd., No. 30 Jinxing Road, Daxing District, Beijing 102600, China

## Abstract

**Background:**

Qingre Lidan tablets (QLTs) are a compound preparation of Chinese medicine that have long been used clinically to treat poor bile circulation caused by the inflammation and obstruction of the gallbladder and bile duct and to relieve jaundice and other symptoms. However, its material basis and mechanism are still unclear. The purpose of this study was to investigate the mechanism and active components of QLTs for treating intrahepatic cholestasis (IHC) in rat models.

**Methods:**

In vivo experiments verified the effect of QLTs on alpha-naphthyl isothiocyanate (ANIT)-induced IHC models in rats. The mRNA and protein expression levels of farnesoid *X* receptor (FXR), bile salt export pump (BSEP), and multidrug-associated protein 2 (MRP2) in the rat liver were detected. UPLC/Q-TOF-MS was used to separate and identify the monomers in QLTs, and a dual-luciferase reporter assay was used to select effective the monomers that stimulate FXR. Among the selected monomers, baicalein was used as a representative to verify the effect on rat IHC models.

**Results:**

QLTs and baicalein significantly reduced the serum biochemical indicators reflecting the changes in liver function among IHC rats and remitted the ANIT-induced liver histopathological changes. The expression levels of FXR, BSEP, and MRP2 in the liver were significantly increased after QLT treatment in a dose-dependent manner. Moreover, six types of active components that activate FXR were selected in QLTs, namely baicalein, wogonin, baicalein II, emodin, dibutyl phthalate, and diisooctyl phthalate.

**Conclusions:**

QLTs and the active component, baicalein, can alleviate IHC in model rats.

## 1. Introduction

Intrahepatic cholestasis (IHC) is a pathophysiological process that damages hepatocytes and the body by disturbing bile secretion and excretion [1]. The clinical symptoms of IHC are excessive accumulation of bile components such as bile acids (BAs), bilirubin, and cholesterol in the liver and systemic circulation. It is often caused by a variety of factors, such as viral hepatitis, hormones, drug induction, and autoimmune diseases [2–5]. Long-term IHC may develop into liver fibrosis and cirrhosis [6, 7]. At present, effective medication options for IHC are limited. There are only two drugs (ursodeoxycholic acid (UDCA) and obeticholic acid (OCA)) approved by the US FDA for the treatment of IHC [8]. OCA is the first drug to be developed in the past two decades for treating patients with IHC who were not responding adequately to UDCA. Unfortunately, many patients experience unbearable pruritus after using it [9, 10]. Since the prevalence of IHC has dramatically increased in recent years, novel drugs are urgently needed. Western medicine against IHC has a characteristically long cycle, high cost, and many adverse reactions. Traditional Chinese medicine has accumulated rich clinical experience in IHC treatment, and its advantages of having a curative effect and fewer side effects have been highly affirmed.

Qingre Lidan tablets (QLTs) possess efficient heat clearing, detoxification, dampness elimination, and jaundice removal properties. Clinically, they are primarily used to treat poor bile circulation caused by inflammation and the obstruction of the gallbladder and bile duct. They are composed of six medicinal herbs, namely *Artemisia capillaris* Thunb (Yinchenhao), *Lysimachia christinae* Hance (Jinqiancao), *Scutellaria baicalensis* Georgi (Huangqin), *Rhei radix et Rhizoma* (Dahuang), *Cortex Magnoliae* Officinalis (Houpo), and *Prunella vulgaris* (Xiakucao). QLTs are made by adding and subtracting the Yinchenhao decoction, and previous studies suggest that Yinchenhao decoction can effectively ameliorate the relevant biochemical indicators in rats with IHC induced by alpha-naphthyl isothiocyanate (ANIT) and can alleviate liver pathological damage. In addition, several studies have demonstrated that choleretic Chinese medicine preparations using Yinchenhao as the core, such as the Yinchenhao decoction and the Yinzhihuang injection, can induce the expression of the liver bile salt export pump (BSEP) and farnesoid *X* receptor (FXR) in model animals [11, 12], and the expression of resistance-related protein 2 (MRP2) and BSEP has a significant correlation with FXR [13]. FXR is a transcription factor that plays a major role in BA homeostasis regulation. Studies have shown that FXR-deficient mice fed a high bile acid diet can exhibit cholestatic liver injury [14]. Given the close relationship of Chinese medicine based on Yinchenhao and FXR, we speculate that QLTs can stimulate FXR and then affect the expression of transporters such as BSEP, which plays a choleretic and hepatoprotective role. Therefore, the transcription and translation levels of FXR, BSEP, and MRP2 transporters will be investigated in this study.

The dual-luciferase reporter assay is currently a common method for analysing the agonistic/antagonistic effects of small molecule compounds on FXR. In this study, UPLC/Q-TOF-MS and dual-luciferase reporter assays were combined to establish a high-throughput separation-analysis-screening platform, which can separate and identify the effective monomers that stimulate FXR to explain the material bases of the liver- and gallbladder-protective effects of QLTs.

## 2. Materials and Methods

### 2.1. Chemicals and Reagents

HPLC-grade methanol and acetonitrile were obtained from Merck (Darmstadt, Germany). Analytical grade formic acid was purchased from Honeywell (NJ, USA). Baicalein and emodin were purchased from the National Institutes for Food and Drug Control (Beijing, China). ANIT, UDCA, and olive oil were obtained from Aladdin (Shanghai, China). QLTs were provided by the Tianjin Nankai Hospital.

### 2.2. QLT Preparation

In the animal experimental design, QLTs were crushed and then dispersed in normal saline to make a suspension solution. For the cell experimental design, QLTs were crushed and then dispersed in distilled water, and the resulting product was fractionated with solvents with escalating polarity, including dichloromethane, ethyl acetate, and n-butanol. The organic solvent was removed with a rotary vacuum evaporator under reduced pressure and then dissolved in ethyl alcohol and water and freeze-dried to obtain organic phase and aqueous phase freeze-dried powders.

### 2.3. Cell Culture

The HL7702 cell line was obtained from the Cell Bank of the Chinese Academy of Sciences (Shanghai, China). The cells were cultured in complete medium (90% DMEM (Gibco, NY, USA) +10% foetal bovine serum (FBS) (Gibco) +1% penicillin/streptomycin (Gibco)) and cultured in a cell incubator at 37°C in 5% CO_2_.

### 2.4. Animal and Experimental Design

A total of 120 male SD rats of SPF grade (200 ± 10 g initial weight) were provided by the Experimental Animal Centre at Tianjin Nankai Hospital (Tianjin, China). The experimental animal licence number is SCXK-(Jun)-2014-0001. The animals were adaptively fed for one week.

Sixty male SD rats were randomly divided into six groups (*n* = 10 per group): the control group, model group, UDCA group (60 mg/kg UDCA), QLT-L group, QLT-M group, and QLT-H group. The QLT dosage in the animal experiments was determined according to the best practice of pharmacological research using the body surface area (BSA) formulas, which is a commonly used drug dosage standard, to calculate the drug dosage in rats. Lastly, combined with the clinical dose of QLTs, we orally administered QLTs at 0.56 g/kg (6.17 times the clinical dose) to the QLT-L group and administered QLTs at 1.12 g/kg and 2.24 g/kg to the QLT-M and QLT-H groups. In addition, 60 male SD rats were randomly divided into six groups (*n* = 10 per group): the control group, model group, UDCA group (60 mg/kg UDCA), baicalein-L group (50 mg/kg baicalein), baicalein-M group (100 mg/kg baicalein), and baicalein-H group (200 mg/kg baicalein). The UDCA group, QLT groups, and baicalein groups were given the above-relevant dose for 7 days, and the control group and the model group were given the same amount of normal saline daily. On the 5th day, all the rats except the control group were given ANIT olive oil solution (60 mg/kg). The control group was given the same amount of olive oil. All dosing was performed intragastrically. Samples were collected and processed 48 h after modelling, and the animals fasted for 12 h before sample collection.

### 2.5. Histopathological Examination

The formaldehyde-fixed liver tissue was paraffin-embedded, stained with haematoxylin and eosin, and then observed under a light microscope (200 times) (Leica, Germany).

### 2.6. Biochemical Analysis

The levels of TBA, TBIL, DBIL, ALT, AKP, and *γ*-GT in rat serum were detected with kits from Nanjing Jiancheng Bioengineering Institute (Nanjing, China).

### 2.7. Total RNA Extraction and Quantitative Real-Time PCR

Total RNA from the liver was extracted with TRIzol according to the manufacturer's instructions (Invitrogen, CA, USA). Total RNA (1 *μ*g) from each sample was reverse transcribed into cDNA using NovoScript® Plus All-in-one 1st Strand cDNA Synthesis SuperMix (Novoprotein, Shanghai, China). RT-PCR was performed using NovoStart® SYBR qPCR SuperMix Plus (Novoprotein) in a 7500 Fast Real-Time PCR system (ABI, CA, USA). The PCR primer sequences are listed in Table 1.

### 2.8. Western Blot Analysis

The total protein was extracted from the liver tissues using prechilled lysis buffer supplemented with PMSF protease inhibitor. The protein samples were fractionated by SDS-PAGE and then transferred to PVDF membranes. The membranes were blocked with 5% nonfat milk and incubated with primary antibodies against *β*-actin (1 : 1000 dilution), FXR (1 : 1000 dilution), BSEP (1 : 3000 dilution), and MRP2 (1 : 2000 dilution) overnight at 4°C. The membranes were incubated with secondary antibodies for 1 h at room temperature. Protein expression was probed with an ECL method (Millipore, USA) and placed in a ChemiDoc XRS System (Bio-Rad, CA, USA).

### 2.9. UPLC/Q-TOF-MS Analysis

Liquid chromatographic separation was performed on a Waters (MA, USA) Acquity UPLC BEH C18 column (2.1 mm × 100 mm, 1.7 *μ*m). Instrument control and data analysis were performed using MassLynx 4.1 software (Waters). The column temperature was 45°C. The flow rate was 0.4 ml/min. The mobile phase consisted of water containing 0.1% formic acid (solvent system A) and acetonitrile (solvent system B), and the gradient elution program was as follows: 0-1 min, 2% B; 1–5.5 min, 2–24% B; 5.5–8.5 min, 24–33% B; 8.5–14.5 min, 33–40% B; 14.5-15.5 min, 40–48% B; 15.5–18 min, 48–54% B; 18–22 min, 54–57% B; 22–30 min, 57–75% B; 30–33 min, 75–79% B; 33-34 min, 79–85% B; 34–36 min, 85–89% B; 36–38 min, 89–100% B; 38-39 min, 100-2% B; and 39-40 min, 2% B. The fraction was collected into cryopreservation tubes (2.0 ml) every 0.5 min for the first 20 min and every 1 min for the next 20 min. The collection of eluent fractions was repeated five times. The collected fractions were evaporated in a vacuum oven at 40°C, and the residues were dissolved in 100 *μ*L of DMEM for the dual-luciferase reporter assay.

The mass data on the ingredients were acquired with a Q-TOF-MS instrument (Waters) with an electrospray ionization (ESI) system operated in positive and negative ion voltage modes. The capillary voltage was kept at 3 kV in positive mode and 2 kV in negative mode. The sample cone voltage was set to 40 V. The source temperature and desolvation temperature were set to 120°C and 400°C, respectively. High-purity nitrogen and argon were chosen as the desolvation gas and the collision gas, respectively, with 50 L/h cone gas flow and 800 L/h desolvation gas flow. The detector voltage was 1900 V, the Q-TOF acquisition rate was 0.1 s, the internal scanning interval delay was 0.02 s, and the mass range was 50–1700 Da. The mass accuracy was previously calibrated with leucine enkephalin in positive ion mode.

### 2.10. Cell Transfection and Luciferase Reporter Assays

The cells were seeded in 24-well plates (1.0 × 10^5^ cells/well) and transfected together with the internal reference luciferase reporter gene plasmid pRL-TK and FXR-luc luciferase reporter gene plasmid pGMFXR-Lu. Twenty-four hours after the transfection, different drugs were added to the wells and incubated. Forty-eight hours after transfection, the cells were harvested and subjected to the dual-luciferase assay (Promega, WI, USA).

### 2.11. Statistical Analysis

SPSS 26.0 software was used for statistical analysis. Continuous variables are represented as the means ± standard deviation ($\bar{x}\pm s$). Statistical differences were evaluated by *t*-test for 2 groups and by one-way analysis of variance (ANOVA) for more than 2 groups. *P* < 0.05 or *P* < 0.01 indicated a significant difference, and *P* > 0.05 indicated no significant difference.

## 3. Results and Discussion

### 3.1. The Attenuating Effects of QLTs against IHC in Animal Models

**Figure 1:**
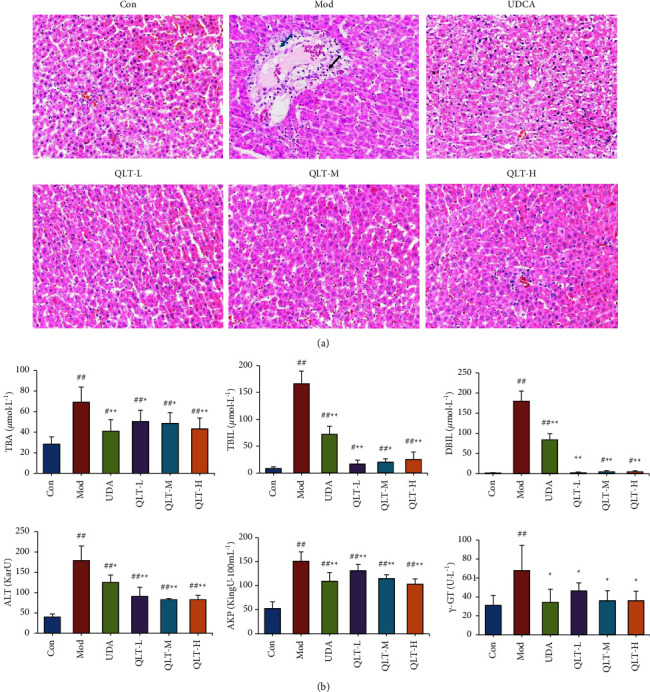
The attenuating effects of QLTs against IHC in animal models. (a). Representative images of HE staining of liver tissue (×200); (black arrow) inflammatory cell infiltration; (blue arrow) loose perivascular connective tissue. (b). Comparison of TBA, TBIL, DBIL, ALT, AKP, and *γ*-GT levels of rats in each group (means ± SD, *n* = 10, compared with the control group, ^#^*P* < 0.05, ^##^*P* < 0.01; compared with the model group, ^*∗*^*P* < 0.05, and ^*∗∗*^*P* < 0.01).

Following induction by ANIT, the rats changed significantly, with changes characterized by mental state deterioration, reduced activity, jaundice symptoms, and yellowing of the sole skin and urine colour. However, the physiological status of the rats treated with UDCA and QLTs was improved to some extent. From the pathological section of liver tissue (Figure 1(a)), we also observed that UDCA and QLTs significantly improved the liver histopathological damage caused by ANIT. In the control group, hepatocytes were arranged radially around the central vein, and the structure of the liver was clearly visible. The hepatocytes were uniform in size, with deeply stained nuclei, intact nuclear membranes, and clearly visible nucleoli. In the model group, the structure of the hepatocytes was clearly deformed, the boundary was unclear, the arrangement of hepatic cords was disordered, the cell nucleus was enlarged, and the boundary was unclear. Congestion of hepatic sinusoids and clusters of inflammatory cell infiltration were observed, indicating successful model replication. Compared with the model group, in the UDCA group and each QLT dose group, the arrangement of hepatocytes was more regular, the cell size was approximately equal, and the oedema phenomenon was significantly improved. Inflammatory cell infiltration was decreased, and degeneration and necrosis of intrahepatic bile duct epithelial cells were also significantly reduced. The hepatic sinusoids and central veins were normal and regular.

At that time, we detected the biochemical indicators of liver function in the serum of rats in each experimental group. Compared with the control group, the levels of TBA, TBIL, DBIL, ALT, AKP, and *γ*-GT were significantly increased in the model group (Figure 1(b)). Furthermore, QLTs attenuated the ANIT-induced stimulation of TBA, TBIL, DBIL, ALT, AKP, and *γ*-GT, indicating the amelioration of liver damage.

### 3.2. QLTs Regulated the FXR-Mediated Bile Acid Pathway to Alleviate CHI

**Figure 2:**
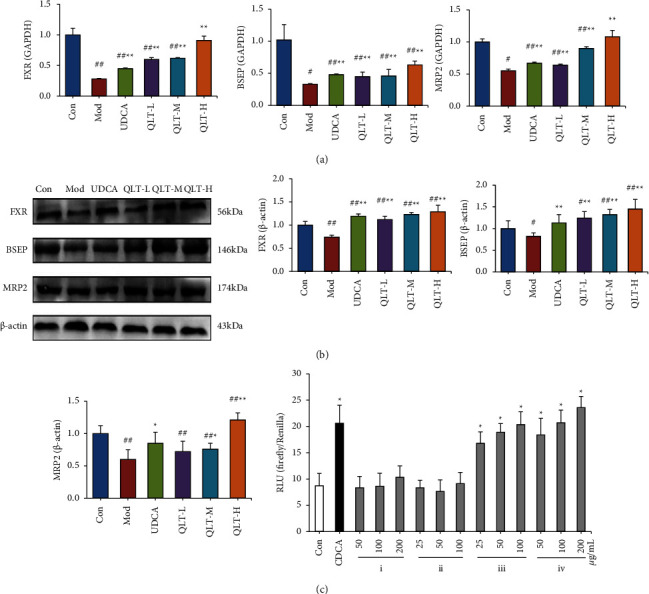
QLTs regulated the FXR-mediated bile acid pathway to alleviate CHI. (a). The effect of QLTs on the relative expression of FXR, BSEP, and MRP2 mRNA in rat liver tissue. (b). The effect of QLTs on the protein expression levels of FXR, BSEP, and MRP2 in the rat liver (means ± SD, *n* = 6, compared with the control group, ^#^*P* < 0.05, ^##^*P* < 0.01; compared with the model group, ^*∗*^*P* < 0.05, ^*∗∗*^*P* < 0.01). (c). Study on activating FXR activity of extracts from QLTs (*n* = 6, compared with control group ^*∗*^*P* < 0.05, (i) water extract, (ii) dichloromethane extract, (iii) ethyl acetate extract, and (iv) n-butanol extract).

Quantitative real-time PCR and western blotting analysis were used to evaluate the related mRNA and protein expression levels in the liver. As shown in Figure 2(a), compared with the control group, the relative mRNA expression of FXR, BSEP, and MRP2 was significantly decreased in the model group (*P* < 0.01). Compared with the model group, the relative mRNA expression of FXR, BSEP, and MRP2 mRNA in the UDCA group and the QLT groups was significantly upregulated (*P* < 0.01). The results suggested that each QLT dose group can significantly upregulate the relative expression of FXR, BSEP, and MRP2 mRNA in liver tissue. Additionally, the results of the protein expression of FXR, BSEP, and MRP2 (Figure 2(b)) showed the same trend as those of mRNA. This result suggested that QLTs regulated the FXR-mediated bile acid signalling pathway to improve ANIT-induced IHC.

FXR response element luciferase assays in HL-7702 cells were performed. As shown in Figure 2(c), CDCA (chenodeoxycholic acid, used as a positive control) and the ethyl acetate and n-butanol extracts of QLTs all increased the luciferase signal intensity, and the increasing trend showed concentration dependence (*P* < 0.05). However, there was no significant difference in the RLU between the water extract and dichloromethane extract of QLTs compared with the control group (*P* > 0.05). Overall, our data suggest that the ethyl acetate extract and n-butanol extract of QLTs contained FXR activators.

### 3.3. Qualitative Study on the Chemical Constituents of QLT Extract

**Figure 3:**
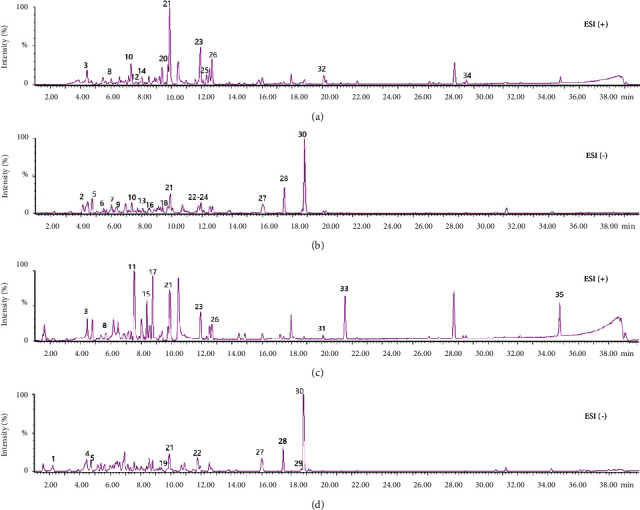
Qualitative study on the chemical constituents of the QLT extract: (a) positive ion mode of the ethyl acetate extract, (b) negative ion mode of the ethyl acetate extract, (c) positive ion mode of the n-butanol extract, and (d) negative ion mode of the n-butanol extract. Mass spectra corresponding to compounds 1–35 are shown in Table 2.

The chemical composition of the active QLT extract (ethyl acetate and n-butanol extract) was qualitatively analysed by UPLC/Q-TOF-MS, and the UPLC/MS positive and negative ion modes of the sample were obtained according to the corresponding chromatographic and mass spectrometry conditions. The result is shown in Figure 3. The figure shows that the peak baseline of the QLT active extract is stable and the peak shape of the target compound is good. The quasimolecular ion peak information of the substance can be obtained through the positive and negative mode analysis of the primary mass spectrum, and then, the fragment ion information in the secondary spectrum is used to analyse the fragmentation law. In the reference literature used for analysis and comparison, a total of 35 chemical components in the ethyl acetate extract and n-butanol extract of QLTs were identified. The identification results of the specific components are shown in Table 2.

### 3.4. Screening and Verification of Active Monomers from QLT-Agitated FXR

**Figure 4:**
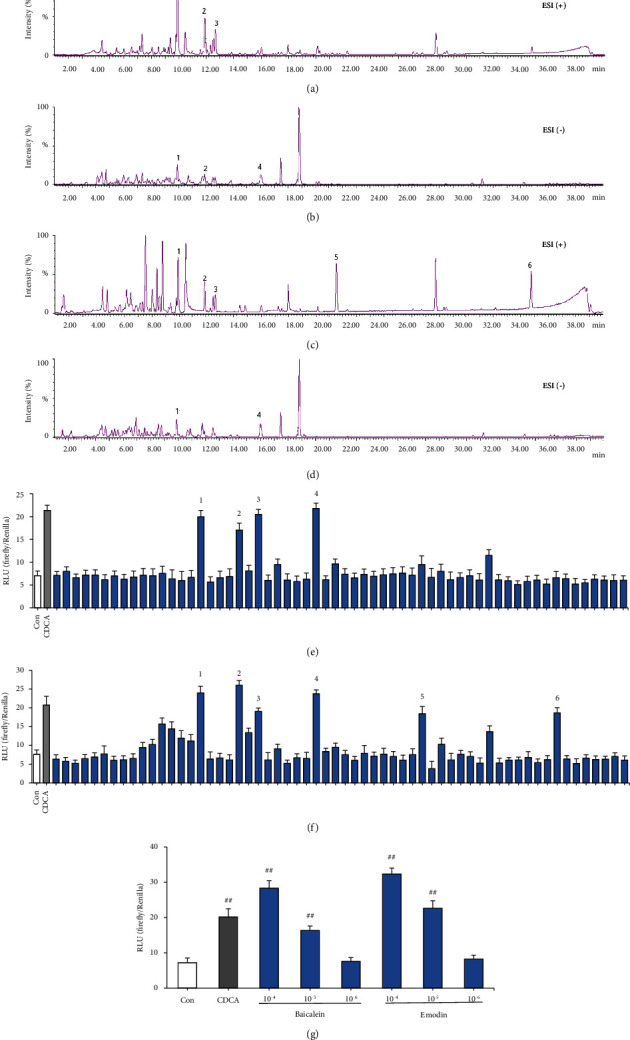
Screening and verification of active monomers from QLT-agitated FXR. (a). Positive ion mode and QLT ester extract; (b). negative ion mode and QLT ester extract; (c). positive ion mode and QLT n-butanol extract; (d). negative ion mode and QLT n-butanol extract; (e). activating activity of each component of QLT ester extract on FXR detected by dual-luciferase reporter gene system; (f). activating activity of each component of alcohol extract of QLTs on FXR detected by dual-luciferase reporter gene system; (g). monomer verification of active ingredients (*n* = 6, ^##^*P* < 0.01 compared with the control group); (1) baicalein, (2) wogonin, (3) skullcapflavone II, (4) emodin, (5) dibutyl phthalate, and (6) diisooctyl phthalate.

Through UPLC/Q-TOF-MS combined with a dual-luciferase reported high-throughput screening system, 6 types of active ingredients that stimulate FXR activity were screened out from ethyl acetate and n-butanol extracts of QLTs, namely baicalein, wogonin, skullcapflavone II, emodin, dibutyl phthalate, and diisooctyl phthalate (Figures 4(a)–4(f)). Then, baicalein and emodin standards were chosen to validate the activation of FXR. As shown in Figure 4(g), the two compounds both had good FXR agonistic activity at 10^–4^ and 10^–5^ mol/L.

### 3.5. Baicalein Protected against IHC Induced by ANIT

**Figure 5:**
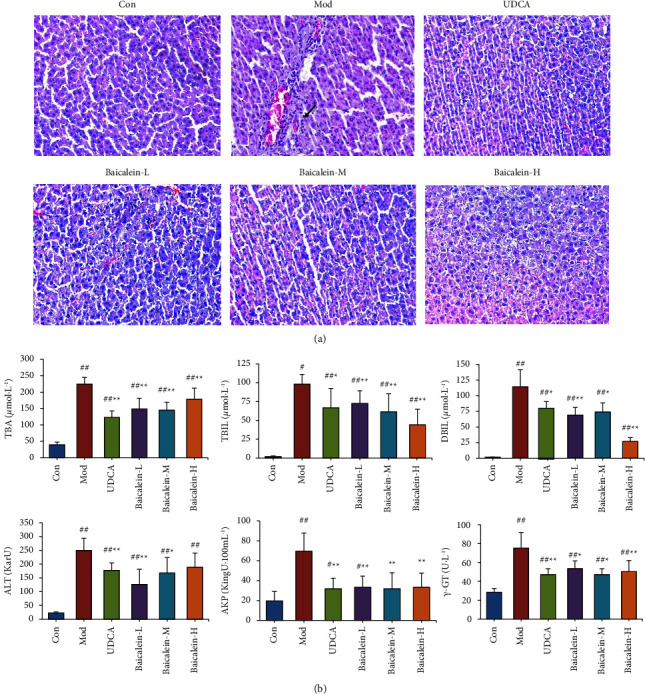
Baicalein protected against IHC induced by ANIT. (a). Effect of baicalein on hepatic histomorphology in rats (HE, ×200); (black arrow) inflammatory cell infiltration. (b). Comparison of serum TBA, TBIL, DBIL, ALT, AKP, and *γ*-GT levels from rats in each group (means ± SD, *n* = 10, compared with the control group, ^#^*P* < 0.05, ^##^*P* < 0.01; compared with the model group, ^*∗*^*P* < 0.05, ^*∗∗*^*P* < 0.01).

To evaluate the pharmacodynamic effect of baicalein, an FXR agonist screened in QLTs, baicalein was used to treat IHC models induced by ANIT. The results showed that this compound can ameliorate the physiological state of model rats and reduce serological indices (Figure 5(a)), including TBA, TBIL, DBIL, ALT, AKP, and *γ*-GT. In addition, the pathological sections of liver tissues in the baicalein group were also significantly ameliorated compared with those in the model group (Figure 5(b)). This observation showed that baicalein had an obvious protective effect on IHC induced by ANIT, which lays a foundation for further research on baicalein.

## 4. Discussion

IHC is a clinical syndrome caused by the accumulation of bile in the liver [28]. Excluding biliary obstruction, it is often induced by viral hepatitis, drug-induced liver damage, genetic factors, and pregnancy [29, 30]. ANIT is a widely known hepatotoxic substance that can induce IHC in rodents. ANIT gavage can cause cholangiocyte damage and the infiltration of inflammatory cells around sinusoids in rats and then lead to the accumulation of a high concentrated gradient of BA and other bile components in the liver [31]. BA is the main component of bile [32]. When ANIT induces IHC, the function of the hepatocytes is impaired, the ability to synthesize, take up, or excrete bile is weakened or disappears, and the bile homeostasis in the body is destroyed, which causes cholate accumulation. Hepatocytes will compensatively excrete bile into the blood to relieve BA accumulation in hepatocytes. Therefore, the TBA and bilirubin in the serum will be higher than those in normal rats. As shown in Figure 1, our results indicated that serum TBA, TBIL, DBIL, ALT, AKP, and *γ*-GT were increased significantly in rats treated with ANIT. In contrast, QLTs ameliorated the levels of TBA, TBIL, DBIL, ALT, AKP, and *γ*-GT and significantly reduced the pathological damage to rat liver tissue. This finding is consistent with the results of previous studies showing that Yinchenhao decoction can effectively relieve IHC and liver pathological damage [33].

FXR is the core regulator of bile acid homeostasis, and it plays a significant role in physiological bile acid synthesis, secretion, and transportation [34]. In recent years, FXR agonists have been widely found to ameliorate cholestasis effectively and are expected to become new drugs for cholestatic liver disease [35, 36]. The main roles of FXR in regulating the transport and synthesis of bile acids are as follows. As a target gene of FXR, the small heterodimer partner (SHP) can be transcribed by activated FXR when intracellular bile acids are elevated. Then, activating the FXR-SHP signalling pathway inhibits the transcription of cholesterol 7-alpha hydroxylase (CYP7A1) and reduces bile acid production [37]. FXR activation in the small intestine can induce the expression of intestinal fibroblast growth factor 15/19 (FGF15/19). When FGF15/19 reaches the liver through the portal vein and activates fibroblast growth factor receptor 4 (FGFR4), the expression of CYP7A1 is reduced, and the synthesis of bile acids is inhibited [38]. FXR can promote the secretion of bile acids by upregulating the expression of BSEP and reducing the concentration of bile acids in the liver [39]. FXR can inhibit the reuptake of bile acids in the liver by downregulating the transcription of sodium taurocholate cotransporting polypeptide (NTCP) to prevent the toxicity resulting from a high concentration of bile acids [39]. In summary, activating FXR can effectively inhibit the accumulation of bile acids in the liver and promote their excretion. Moreover, studies have reported that the hepatoenteric circulation of bile acids is regulated by FXR and many special transporters, among which BSEP and MRP2 are involved in this process [40]. MRP2 and BSEP are target genes of FXR and are directly induced by FXR at the transcriptional level [13, 41]. BSEP is the rate-limiting step and main transporter and is responsible for the secretion of bile salt from the bile duct lateral membrane of hepatocytes [42]. Its gene mutation can cause BSEP to lose its ability to transport and bind cholate [37], which is manifested as a disorder of bile secretion, which in turn leads to IHC. The MRP2 protein is not only responsible for transporting bilirubin from cells to the bile duct but also participates in the transport of a variety of hepatotoxic substances and protects hepatocytes [43]. In our study, ANIT inhibited the expression of FXR, BSEP, and MRP2, which was consistent with previous findings [13, 44], and QLTs could reverse this trend (Figure 2), suggesting that QLTs could alleviate ANIT-induced liver injury.

After clarifying the agonistic effect of QLTs on FXR, we wanted to investigate the material basis by which QLTs alleviated IHC. We integrated UPLC/Q-TOF-MS with a dual-luciferase reporter gene assay to separate and screen effective monomers with FXR agonists active in QLTs. The results showed that baicalein, wogonin, skullcapflavone II, emodin, dibutyl phthalate, and diisooctyl phthalate in QLTs have obvious FXR agonistic activity (Figure 4). In previous studies, baicalein alleviated acute liver injury and hepatocyte oxidative stress by upregulating antioxidant defence pathways and downregulating oxidative stress, apoptosis, and inflammation [45–47]. Wogonin can alleviate liver injury and liver fibrosis by regulating oxidative stress, the inflammatory response, and the activation and apoptosis of hepatocytes [48, 49]. Many studies have indicated that emodin promotes bile excretion by stimulating the FXR/BSEP pathway to ameliorate ANIT-induced IHC [50, 51]. To verify whether the above monomers can ameliorate IHC, we selected one of the monomers as a representative for in vivo pharmacodynamic evaluation. Combined with UPLC/Q-TOF-MS technology, we found that baicalein had high content and good activity in the ethyl acetate and n-butanol extracts of QLTs. We ultimately selected this compound to study the pharmacodynamics in IHC model rats. Baicalein, a flavonoid, is one of the most important active components of Huangqin. Studies have shown that baicalein can alleviate liver injury, which is consistent with our results [46]. However, in the present study, baicalein was not as effective as QLTs in ameliorating the serological indices and liver pathology of cholestasis model rats, probably due to the different amounts of baicalein in QLTs, because only qualitative research was performed by UPLC/Q-TOF-MS. In addition, the reason may be related to the overall regulation of multicomponent, multichannel, and multitarget effects of QLTs.

## 5. Conclusions

Our results show that QLTs and the active component, baicalein, have an obvious protective effect on IHC induced by ANIT in rats. These effects were mediated by activating the FXR signalling pathway and upregulating the expression of BSEP and MRP2 transporters, promoting bilirubin transport and bile acid excretion.

## Figures and Tables

**Table 1:** Primers for quantitative real-time PCR.

Gene name	Nucleotide sequence		Amplicon size (bp)
FXR	Sense	CAGCAGACCCTCCTGGATTA	140
	Antisense	ACGTGACTGGTAGCCATTTC	

BSEP	Sense	TGCTTATGGGAGGCGTAT	565
	Antisense	GGGCTGACAGCAAGAATC	

MRP2	Sense	GGCTGAGTGCTTGGAC	789
	Antisense	CTTCTGACGTCATCCTCAC	

GAPDH	Sense	AGATGGTGAAGGTCGGTGTG	230
	Antisense	CTGGAAGATGGTGATGGGTT	

**Table 2:** Chemical constituent identification of QLTs.

No.	tR/min	Measured (*m*/*z*)	Calculated (*m*/*z*)	Mode	Molecular formula	Error (ppm)	Fragment (*m*/*z*)	Name	Source	Reference
1	1.23	169.0123	169.0137	b, [M − H]^−^	C_7_H_6_O_5_	−8.3	125, 97, 79	Gallic acid	RRR	[15]
2	3.01	137.0234	137.0239	a, [M − H]^−^	C_7_H_6_O_3_	−3.6	137, 93	Salicylic acid	SBG; ACT	[15]
3	3.44	291.0876	291.0869	b, [M + H]^+^	C_15_H_14_O_6_	2.4	139, 123, 91	(−)-Epicatechin	RRR	[15]
4	3.68	353.0856	353.0873	b, [M − H]^−^	C_16_H_18_O_9_	−4.8	191, 179	Chlorogenic acid	ACT; LCH	[16]
5	3.73	179.0332	179.0344	a, [M − H]^−^	C_9_H_8_O_4_	−6.7	179, 135	Caffeic acid	ACT; LCH; PV	[16]
6	4.50	163.0383	163.0395	a, [M − H]^−^	C_9_H_8_O_3_	−7.4	163	p-Coumaric acid	ACT; LCH; PV	[17]
7	4.90	301.0352	301.0348	a, [M − H]^−^	C_15_H_10_O_7_	1.3	301, 289, 125	Quercetin	ACT; LCH	[18]
8	4.96	611.1611	611.1612	b, [M + H]^+^	C_27_H_30_O_16_	−0.2	633, 611, 303	Rutin	ACT; PV; LCH	[18]
9	5.55	447.0930	447.0927	a, [M − H]^−^	C_21_H_20_O_11_	0.7	285, 227	Kaempferol-3-O-galactoside	LCH	[19]
10	6.35	347.0778	347.0767	a, [M + H]^+^	C_17_H_14_O_8_	3.2	332, 317, 314	Ganhuangenin	SBG	[20]
11	6.54	447.0942	447.0927	b, [M + H]^+^	C_21_H_18_O_11_	2.0	271, 253	Baicalin	SBG	[15]
12	6.87	303.0504	303.0505	a, [M + H]^+^	C_15_H_10_O_7_	−0.3	303, 285, 257, 229	Morin	ACT; PV	[21]
13	7.03	461.1079	461.1084	a, [M − H]^−^	C_22_H_22_O_11_	−1.1	331, 169	LMPK12111078^*∗*^	SBG	[15]
14	7.05	177.0560	177.0552	a, [M + H]^+^	C_10_H_8_O_3_	4.5	177, 163	7-Methoxycoumarin	ACT	[21]
15	7.38	461.1088	461.1084	b, [M + H]^+^	C_22_H_20_O_11_	0.9	285, 270	Oroxylin A-7-O-glucuronide	SBG	[15]
16	7.59	431.0971	431.0978	a, [M − H]^−^	C_21_H_20_O_10_	−1.6	431, 269	Emodin-8-O-*β*-D-glucopyranoside	RRR	[20]
17	7.77	461.1090	461.1084	b, [M + H]^+^	C_22_H_20_O_11_	1.3	285, 270	Wogonoside	SBG	[15]
18	8.38	269.0446	269.0450	a, [M − H]−	C_15_H_10_O_5_	−1.5	269, 253, 241, 225	Aloe emodin	RRR	[15]
19	8.73	241.0862	241.0865	b, [M − H]^−^	C_15_H_14_O_3_	−1.2	241, 223, 182	Randaiol	CMO	[22]
20	8.78	301.0714	301.0712	a, [M + H]^+^	C_16_H_12_O_6_	0.7	286	4′-Hydroxywogonin	SBG	[23]
21	8.90	271.0620	271.0606	a, [M + H]^+^	C_15_H_10_O_5_	5.2	253, 241	Baicalein	SBG	[15]
22	10.75	283.0252	283.0243	a, [M − H]^−^	C_15_H_8_O_6_	3.2	282, 239, 211, 183	Rhein	RRR	[15]
23	10.93	285.0771	285.0763	a, [M + H]^+^	C_16_H_12_O_5_	2.8	270, 151	Wogonin	SGB	[15]
24	11.10	253.0506	253.0501	a, [M − H]^−^	C_15_H_10_O_4_	2.0	253, 225	Chrysophanol	RRR	[15]
25	11.33	315.0875	315.0869	a, [M + H]+	C_17_H_14_O_6_	1.9	315, 154	Cirsimaritin	SBG; ACT	[15]
26	11.68	375.1087	375.1080	b, [M + H]+	C_19_H_18_O_8_	−1.9	360, 345	Skullcapflavone II	SBG	[15]
27	15.00	269.0448	269.0450	a, [M − H]^−^	C_15_H_10_O_5_	−0.7	269, 253, 241, 225	Emodin	RRR	[15]
28	16.48	265.1239	265.0229	a, [M − H]^−^	C_18_H_18_O_2_	3.8	265, 235, 223, 197	Honokiol	CMO	[22]
29	17.68	281.1164	281.1178	b, [M − H]^−^	C_18_H_18_O_3_	−5.0	281, 263, 133	Randainol	CMO	[24]
30	17.82	265.1246	265.1229	a, [M − H]^−^	C_18_H_18_O_2_	6.4	265, 247, 223	Magnolol	CMO	[22]
31	19.10	285.0771	285.0763	b, [M + H]^+^	C_16_H_12_O_5_	2.8	270	Oroxylin A	SBG	[15]
32	19.25	279.2321	279.2324	a, [M + H]^+^	C_18_H_30_O_2_	−1.1	279, 119, 105, 95	Linolenic acid	ACT	[21]
33	20.54	279.1599	279.1596	b, [M + H]^+^	C_16_H_22_O_4_	1.1	149	Dibutyl phthalate	ACT; CMO	[25]
34	28.49	281.2475	281.2481	a, [M + H]^+^	C_18_H_32_O_2_	−2.1	264, 184	Linoleic acid	PV	[26]
35	34.76	391.2849	391.2868	b, [M + H]^+^	C_24_H_38_O_4_	0.3	149	Diisooctyl phthalate	SBG	[27]

(a) Ethyl acetate extract of QLTs; (b) n-butanol extract of QLTs. (LMPK12111078^*∗*^) 5,7,2′-Trihydroxy-6-methoxyflavone 7-O-glucoside. (ACT) *Artemisia capillaris* Thunb, (LCH) *Lysimachia christinae* Hance, (SBG) *Scutellaria baicalensis* Georgi, (RRR) *Rhei radix et Rhizoma*, (CMO) *Cortex Magnoliae* Officinalis, (PV) *Prunella vulgaris*.

## Data Availability

The data used to support the findings of this study are available from the corresponding author upon request.
